# Skeletal endocrinology: where evolutionary advantage meets disease

**DOI:** 10.1038/s41413-021-00149-x

**Published:** 2021-05-28

**Authors:** Nikolai Jaschke, Wolfgang Sipos, Lorenz C. Hofbauer, Tilman D. Rachner, Martina Rauner

**Affiliations:** 1 Department of Medicine III & Center for Healthy Aging, Technische Universität Dresden, Dresden, Germany; 2 Clinical Department for Farm Animals, University of Veterinary Medicine Vienna, Vienna, Austria

**Keywords:** Metabolic bone disease, Bone

## Abstract

The regulation of whole-body homeostasis by the skeleton is mediated by its capacity to secrete endocrine signaling molecules. Although bone-derived hormones confer several adaptive benefits, their physiological functions also involve trade-offs, thus eventually contributing to disease. In this manuscript, we discuss the origins and functions of two of the best-studied skeletal mediators, fibroblast growth factor 23 and osteocalcin, in an evolutionary context. Moreover, we provide a theoretical framework seeking to explain the broad involvement of these two hormones in amniote physiology as well as their potential to fuel the development and progression of diseases. Vice versa, we outline which perturbations might be amenable to manipulation of these systems and discuss limitations and ongoing challenges in skeletal endocrine research. Finally, we summarize unresolved questions and potential future studies in this thriving field.

## The evolution of bone

Over 540 million years ago, life on earth changed dramatically within a relatively short period of time (i.e., roughly 20 million years) known as the Cambrian explosion. This epoch was characterized by the appearance of multiple novel species with distinctive features such as skeletons, whereas skeleton-like, body-forming and stabilizing structures had evolved even earlier.^1–3^ While the processes driving Cambrian explosion still remain incompletely understood, several possible explanations for the appearance of skeletons as we know them today exist. Some non-vertebrates (i.e., primarily the arthropods) developed shells (exoskeletons) composed of calcium carbonate (CaCO_3_)-deposits embedded into the cuticular chitin-arthropodin matrix, which proved beneficial as a protection against external trauma elicited by predators.^2,4^ Moreover, these structures provided insertion points for the musculature, thus enabling targeted and fine-tuned locomotion. In addition, the shield-like properties of such exoskeletons might have aided in osmoregulation by lowering constant freshwater exposure of the host.^5^ Therefore, exoskeletons conferred an important survival advantage and led to the development of larger organisms. However, their rigidity also restricted growth beyond a certain point (several cm with very few exceptions) and confined mobility to some extent.^3^

The evolution of the vertebrate endoskeleton, on the other hand, was an important step in the phylogeny of endoskeletons, as it relied on hydroxyapatite (Ca_5_[OH(PO_4_)_3_]) as the main non-organic building block, which conferred several possible advantages.^6^ In addition to the aforementioned role in locomotion, vertebrate endoskeletons offered protection of the vulnerable central nervous system and later on, also of inner organs such as the lung and heart. Likewise, hydroxyapatite-based vertebrate endoskeleton gave rise to another major function of modern bone, namely the participation in the regulation of mineral homeostasis. Indeed, vertebrate endoskeletons serve as a rich depot for both calcium and phosphate.^5^ Notably, all cellular processes (and thus, life) are ultimately dependent on phosphate (_Pi_) given its central role in energy metabolism (i.e., high-energy pyrophosphate bonds of ATP).^7^ Hence, hydroxyapatite-based endoskeletons provide the most basic building block to produce energy, which is especially crucial to animals with high-energy requirements as found in vertebrates.

Another potential reason for the advantage of hydroxyapatite could lie in its increased acid-resistance. Vertebrates typically exhibit periods of bursting activity (e.g., hunting), where anaerobic glycolysis is utilized to meet the host’s energy demands. However, this metabolic program yields lactic acid as an endproduct and may elicit a decrease of extracellular pH. As such, the higher pH-resistance of hydroxyapatite compared to calcium carbonate would render bone less susceptible to skeletal dissociation, therefore reflecting an adaptive trait.^8^ Finally, a bone apparatus suitable for drastically enhanced mechanical loading going in parallel with terrestrial life also enabled the evolution of larger species.^3^

Collectively, these features of the hydroxyapatite endoskeleton imposed the risk of transient and/or permanent accumulation of phosphate within the body, which is highly toxic and evokes diseases such as vascular calcification.^9^ Accordingly, the transition from all-calcium to phosphate-based skeletons required the development of a sensitive system to maintain homeostasis.

### Maintenance of homeostasis by the skeleton

Homeostasis describes how certain variables are maintained within a predefined range through regulatory mechanisms. Typically, a sensor detects the current state of a given variable to compare it with a predefined desired range. If the status quo and the set point are not consistent, homeostatic mechanisms are prompted to restore the latter.^10^ These counterregulatory programs may be exerted by various tissues and organs but most of them heavily rely on endocrine mediators. Over the past years, it has become apparent that bone actively participates in maintaining homeostasis in amniotes (clade of four-limbed vertebrates encompassing birds, reptiles and mammals) through secreting endocrine signaling molecules as exemplified by the central role of the highly sensitive fibroblast growth factor 23 (FGF23) system in phosphate metabolism. While bone-derived endocrine signaling molecules confer several evolutionary benefits, they also come at the cost of increased vulnerability to disease, especially under rapidly changing environmental conditions. In the following sections, we will discuss two prominent skeletal hormones, FGF23 and osteocalcin (OCN), in light of evolution and the respective benefits and trade-offs of their implementation.

## Fibroblast growth factor 23 (FGF23)

FGF23 is mainly produced by osteocytes, although a variety of cellular sources have been described in the literature comprising macrophages, cardiomyocytes, enterocytes or epithelial cells in the kidney, among others.^11–15^ Circulating FGF23 levels are regulated by several means including transcriptional as well as posttranscriptional mechanisms. Importantly, intact FGF23 (iFGF23) can be cleaved by a hitherto unknown (furin-like) protease, thus yielding N- and C-terminal fragments (nFGF23 and cFGF23, respectively), which may exhibit distinct biological functions that differ from those of the intact molecule.^16^

### FGF23 biology

Human FGF23 is a 32 kDa glycoprotein built from 251 amino acids with 72% and 71% homology to rat and mouse FGF23, respectively.^17^ Multiple molecules have been shown to regulate FGF23 transcription including DMP1, PHEX, Vitamin D, pro-inflammatory cytokines, PTH, aldosterone as well as phosphate.^11,18–22^ A highly conserved subtilisin-like proprotein convertase cleavage site separates the N- and C-terminal domain of FGF23. Notably, this motif is unique to FGF23 and not present in any other FGF family members.^23^ To date, the protease facilitating FGF23 cleavage has not been identified. Mutations within this amino acid sequence render the molecule cleavage resistant, thus yielding an accumulation of iFGF23. This iFGF23 excess culminates in disease, namely autosomal dominant hypophosphatemic rickets (ADHR).^24^ Indeed, the posttranslational modification of iFGF23 is highly complex and only partly understood. Polypeptide N-acetylgalactosaminyltransferase 3 (GalNT3) protects the full-length hormone from being cleaved by O-glycosylation at Thr^178^, whereas phosphorylation at Ser^180^ via the secretory protein kinase family with sequence similarity 20 (FAM20C) interferes with this process. As such, GalNT3 favors iFGF23 production, whereas FAM20c promotes cFGF23 secretion. Both FGF23 forms are readily detectable in the circulation but the relative proportions may drastically change, especially in disease (e.g., CKD or anemia).^25^ Which functions cFGF23 fulfills remains obscure but some studies have suggested that it antagonizes classical iFGF23 signaling.^26^

Intact FGF23 (iFGF23) binds to FGFRs with various affinity through its C-terminal domain, while the N-terminal domain interacts with the prototypical FGF23 co-receptor alpha Klotho.^27^ The longstanding belief that FGF23 exclusively acts on organs that highly express alpha Klotho has recently been challenged by studies showing that FGF23 also elicits alpha Klotho independent effects (e.g., via FGFR4).^28^

**Fig. 1:**
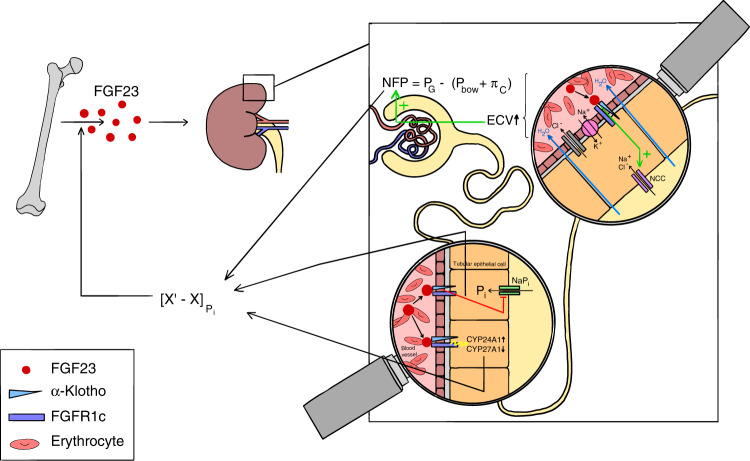
Homeostatic functions of FGF23. Serum levels of phosphate are maintained within a tight range ([X′-X]) through constantly comparing the status quo (X) of circulating phosphate abundance with a predefined set-point (X′). If phosphate levels rise above the upper boundary of this range, FGF23 is induced, although the precise mechanism underlying phosphate-driven FGF23 secretion remains elusive. FGF23 restores phosphate homeostasis through its effects on the kidneys: In the proximal tubule, FGF23 reduces phosphate reabsorption by downregulating Natrium/Phosphate Cotransporters (NaP_i_s), inhibition of 25-OH-Vitamin D activation through downregulating CYP27A1 as well as enhanced catabolism of 25-OH-Vitamin-D via upregulation of CYP24A1. The latter two changes also decrease intestinal phosphate uptake. Moreover, FGF23 enhances sodium and chloride reabsorption in the distal tubule by fostering Na^+^/Cl^−^ Cotransporter (NCC) activity, which yields an expansion of extracellular volume (ECV). This, in turn, increases systemic as well as glomerular blood pressure (P_G_), thereby enhancing net glomerular filtration pressure (NFP) and thus, phosphate filtration. The two opposing forces of P_G_ restricting NFP are the hydrostatic pressure within the capsular of Bowman (P_Bow_) and glomerular capillary oncotic pressure (π_c_), i.e., [NFP = P_G_-(P_Bow_ + π_c_)]. Collectively, these adaptations contribute to the phosphate-lowering effects of FGF23

FGF23 plays a central role in phosphate homeostasis through pleiotropic effects that all aim at lowering circulating phosphate abundance. Mechanistically, FGF23 reduces the brush border expression of Na^+^/_Pi_ cotransporters (NaP_i_s) in the proximal tubule and restrains 1-alpha-hydroxylation (i.e., activation) of 25-Hydroxy-Vitamin D. Simultaneously, the catabolism of the 25-Hydroxy-Vitamin D is enhanced through upregulation of CYP24A1. Collectively, these changes promote phosphate excretion by the kidneys, while intestinal phosphate uptake is attenuated (Fig. 1).^22^ The fact that a highly sensitive phosphate-lowering hormone arose in parallel with hydroxyapatite endoskeletons during evolution suggests that accumulation of phosphate should be avoided at high (if not all) cost.^5^ Indeed, levels of phosphate are inversely correlated to life span among different species.^29^ Significantly, hydroxyapatite-based skeletons in the absence of a regulatory FGF23 system have not been reported in gnathostomes (jawed vertebrates), reinforcing the notion that unrestricted phosphate accumulation is not compatible with life. Finally, Klotho knock-out mice, which are deficient in the prototypical FGF23 co-receptor alpha Klotho, exhibit signs of premature aging including typical age-associated diseases such as osteoporosis, impaired glucose tolerance and hearing loss in the presence of excessive phosphate serum levels.^30^ Taken together, tight control of systemic phosphate abundance is indispensable to vertebrate physiology.

### Why does FGF23 regulate hemodynamics?

Additional research has revealed functions of FGF23 that might appear unrelated to phosphate homeostasis at the first glance. This can be readily demonstrated by the reciprocal interactions between FGF23 and the Renin-Angiotensin-Aldosterone-System (RAAS). RAAS is the major regulator of fluid (i.e., extracellular volume) homeostasis and its activity is primarily governed through volume sensing of specialized (juxtaglomerular) epithelial cells localized in the vasa afferentia of the kidneys. The two main effectors of RAAS, angiotensin II and aldosterone, foster sodium and water reabsorption when organ perfusion is insufficient, thereby increasing blood pressure to restore homeostasis.^31^ Remarkably, similar effects have been described for FGF23. Both, the expression as well as the activity of Na^+^/Cl^−^ cotransporter (NCC) in the distal tubule are induced by FGF23.^32^ These functions contribute to phosphate homeostasis because (a) excretion of the vast majority of substances found in terrestrial vertebrates requires prior filtration through the glomerulus, (b) this filtration capacity is primarily regulated by the modulation of intraglomerular blood pressure and (c) an increase in glomerular pressure will result in enhanced filtration and ultimately excretion of a given molecule.^33^ As such, ensuring sufficient renal perfusion and filtration pressure is crucial for FGF23 to fulfill its main biological function (i.e., phosphate excretion). Acutely, the expansion of extracellular volume induced by FGF23 may also “dilute” circulating phosphate levels, followed by the restoration of a physiological volume status through pressure diuresis and suppression of RAAS activity.^32^ Indeed, preclinical studies have shown that hypovolemia-triggered RAAS activation fosters FGF23 expression in bone, which, in turn, increases extracellular volume and blood pressure through induction of NCC activity. Together these changes yield a suppression of aldosterone levels, thus forming a negative feedback loop to avoid pathological volume overload.^34^ In line with this notion, mice overexpressing membrane-bound alpha Klotho exhibit elevated circulating levels of FGF23 and suppressed aldosterone levels. However, these animals develop hypertension suggesting that enhanced expression of FGF23 itself is sufficient to disrupt blood pressure regulation and evoke disease independent of RAAS.^35^

Recently, FGF23 has been linked to the development of anemia through its effects on erythropoietin production, erythroid cell apoptosis and cell cycle regulation.^36^ Similar to blood pressure modulation, these functions of FGF23 may appear to serve other purposes than maintaining phosphate homeostasis but hemodynamics could again help to explain how these effects favor the molecule’s phosphaturic effects. The risk of thromboembolism is directly correlated to hematocrit levels (i.e., blood viscosity).^37,38^ Since erythrocytes are the major determinants of hematocrit levels, an increase in red blood cell count will inevitably enhance the risk of clotting. This is especially evident in pathologies associated with unrestricted erythroid lineage proliferation such as polycythemia vera.^39^ Importantly, highly perfused organs and small vessel networks are especially prone to thromboembolism and associated perfusion defects. This explains why glomerular vessels (and hence, kidney function) are commonly affected by thromboembolic events.^40^ Therefore, the negative regulation of erythropoiesis by FGF23 may serve in lowering blood viscosity to avoid unfavorable hemodynamics within the glomerulus in times of increased risk of clotting to ensure sufficient kidney perfusion and consequently, phosphate excretion. However, persistent activation of these pathways results in disease, namely anemia.^36^

### Cost-benefit considerations of FGF23 functions

One of the best-studied pathologies associated with overactivated FGF23 signaling is chronic kidney disease (CKD), the latter being defined by persistent (>3 months) kidney dysfunction (GFR <60 mL·min^−1^ per 1.73 m^2^). Here, FGF23 levels rise in early stages of the disease and continue to increase with progressive nephron destruction.^41^ When nephrons reach their maximum phosphate excretory capacity, further FGF23 production is futile given that NaP_i_ expression cannot be further downregulated. On the other hand, increasing extracellular volume and blood pressure partly circumvents this problem due to glomerular hyperfiltration as outlined above.

Notably, hyperaldosteronism is a common feature of CKD.^42^ Therefore, FGF23-induced volume expansion in CKD does not suppress RAAS activity but rather contributes to a vicious cycle of volume overload, blood pressure increase, glomerular hyperfiltration and ultimately, nephron loss. Pertinent to this, FGF23 levels are associated with progression of CKD to end-stage renal disease, independent of traditional risk factors such as proteinuria.^43^ Moreover, glomerular hyperfiltration is a common mechanism in the pathogenesis of many kidney diseases including diabetic nephropathy^44^ and vice versa, pharmacological reduction of glomerular pressure (partly) protects against disease progression and reduces mortality.^45–47^

**Fig. 2:**
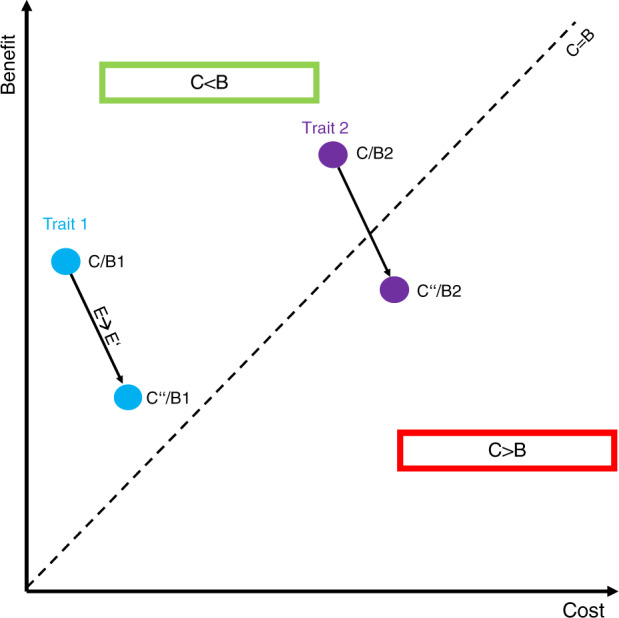
Environmental changes alter cost-benefit ratios. Every trait is characterized by a cost-benefit ratio (C/B) that is confined to a given environment. Trait 1 (blue) is a moderate benefit, low cost trait (C/B1), while Trait 2 (purple) exhibits a high cost, high-benefit ratio (C/B2), the latter being sensitive to environmental factors. Upon a given environmental change (E→E′), Trait 1 thus remains adaptive (C < B, as in C”/B1), while the same change (note the identical length of the black arrow) renders Trait 2 maladaptive (C > B as in C”/B2) (after^48^)

As such, the benefits of FGF23-driven volume regulation to balance phosphate levels in CKD come at the cost of progressive kidney damage, which reflects an unfavorable trade-off, i.e., the cost is higher than the benefit (Fig. 2). From an evolutionary perspective, an adaptive trait is characterized by a cost that is lower than its benefit (C/B < 1). Importantly, this ratio is always confined to a given environment. Thus, rapidly changing environmental conditions may render a trait useless or even detrimental (i.e., maladaptive, C/B > 1) (Fig. 2).^48^ For the vast majority of amniote evolution, a highly sensitive FGF23 system primed to avoid phosphate overloading has been adaptive (i.e., C/B < 1). However, with the increasing prevalence of CKD-inducing pathologies such as diabetes mellitus or hypertension in today’s society^49,50^ that may persistently trigger FGF23 production, the cost of some FGF23-related functions has exceeded their benefit, thus culminating in disease. Another example for such an unfavorable trade-off in FGF23 biology is left ventricular hypertrophy.^51^ Similar to volume expansion, cardiomyocyte hypertrophy is effective in enhancing cardiac output transiently but may ultimately result in heart failure over the long run (C/B > 1).^52^ As such, FGF23 might represent a promising pharmacological target to attenuate pathological changes elicited by its excessive overproduction. On the other hand, blockade of FGF23 could accelerate phosphate retention within the body and consequently, vascular disease.

Here, it is worth noting that somewhat counterintuitive concepts have often yielded remarkable results in human pharmacotherapy as exemplified by beta blockers. In chronic heart failure, where cardiac output is already impaired, inhibition of one of the main pathways promoting cardiomyocyte function (i.e., sympathetic beta receptors) would be expected to worsen the course of the disease. However, this approach disrupts a self-sustaining vicious cycle, reduces mortality and is now well-established as one of the first line therapies of this disease.^53^ Similarly, anti-FGF23-based therapies may prove useful in the treatment of CKD despite their potential phosphate-retaining effects. Future studies may resolve the question whether the benefit of such therapies is indeed higher than their cost.

Remarkably, FGF23 serum levels are also linked to incident hypertension in the general population independently of kidney function suggesting that other factors drive its overproduction in modern society.^54,55^

Two behaviors that substantially differentiate us from our ancestors are lack of physical activity and drastically changed dietary patterns. In fact, industrialized foods frequently contain high levels of phosphate and may thus reflect an important driver of FGF23 production (and FGF23-driven diseases) in our society.^56^ In line with this notion, high phosphate diets stimulate FGF23 production^57^ and low dietary sodium intake potentiates the effect of FGF23 on volume regulation,^32^ suggesting that dietary variables are indeed sufficient to alter FGF23 biology. Lastly, the FGF23 system is a high-cost, high-benefit trait (as is inflammation), which are usually most susceptible to environmental changes (as shown in Fig. 2),^48^ reinforcing the notion that factors of modern society may contribute to FGF23-driven diseases.

### FGF23 and iron metabolism—an unresolved conundrum

One of the multiple unanswered questions in FGF23 research is the molecule’s involvement in iron homeostasis. Iron deficiency induces FGF23 expression which is paralleled by enhanced posttranslational cleavage of the intact molecule, ultimately yielding increased circulating levels of cFGF23, while iFGF23 abundance remains largely unaltered.^58–60^ Notably, the same holds true for pro-inflammatory stimuli.^19,21^ To date, it has not been unraveled which function these changes fulfill and some authors have even questioned that cFGF23 elicits biological effects. On the other hand, it seems implausible that a specific biological program is initiated without serving a purpose. This notion is corroborated by the highly conserved nature of the amino acid motif for FGF23 cleavage among different species suggesting that FGF23 fragments might hold distinctive roles in vertebrate physiology. Since neither C-, nor N-terminal FGF23 evoke phosphaturic effects, posttranslational FGF23 processing adds another layer of complexity to its biology.^16,61^ Remarkably, some studies have suggested that cFGF23, in fact, antagonizes signaling pathways induced by the intact molecule, thereby inhibiting phosphate excretion.^26^ This could imply that transient phosphate retention might be favorable in the presence of pathologies whose correction impose a high energetic burden, i.e., restoration of anemia or fighting invading pathogens through inflammatory responses. Under these circumstances, the cost of phosphate retention is presumably lower than its benefit given that both severe anemia and infection constitute life-threatening conditions.

On the other hand, disrupting these fine-tuned mechanisms elicits unfavorable effects. For example, pharmacological interventions interfering with FGF23 post-translational processing entail hypophosphatemia and associated clinical symptoms.^62^ This has been especially well-documented for certain iron formulations such as ferric carboxymaltose (FCM). Randomized clinical trials have revealed that ~75% of patients with iron deficiency receiving FCM develop hypophosphatemia (circulating phosphate <2 mg•dL^–1^) compared to only 8% of individuals treated with iron isomaltoside.^63^ Within 24 h after FCM injection, patients exhibited a dramatic increase in iFGF23 levels (46–151 pg•mL^–1^), which peaked at even higher levels after the second injection. These changes were associated with renal phosphate wasting, reduced circulating 1,25-dihydroxycholecalciferol levels as well as secondary hyperparathyroidism, all of which indicate FGF23 excess. To date, the precise molecular mechanisms mediating these dysregulations remain obscure, which is partly attributable to the incomplete understanding of post-translational FGF23 processing in physiology (see above). Thus, future research addressing the roles of FGF23 fragments will help to unravel the true functions of these peptides in health and disease.

## Osteocalcin

Osteocalcin (OCN) is the most abundant, non-collagenous protein of bone matrix and mainly synthesized by osteoblasts. Built from 46 to 50 amino acids, OCN undergoes posttranslational modifications such as vitamin K-dependent gamma-carboxylation, which critically contribute to its functions within the skeleton (e.g., through conferring high calcium binding affinity).^64^ Given the inherently high expression of carboxylated OCN in bone, it would be reasonable to assume that genetic deletion of OCN provokes strong skeletal phenotypes. In contrast, most studies have found only mild alterations in OCN knock-out animals characterized by enhanced, rather than reduced bone mass, likely due to increased bone formation, suggesting a negative regulatory role of the protein in skeletal homeostasis.^65,66^ In addition, an inhibitory role of OCN in bone mineralization has been suggested although published data does not uniformly support this concept.^67–70^

In contrast to carboxylated osteocalcin, undercarboxylated OCN (unOCN) has less affinity for bone and its embedded minerals, thus acting as an endocrine hormone exerting a broad spectrum of biological effects in mammals. As such, unOCN regulates insulin sensitivity and glucose homeostasis, promotes male fertility, fosters memory and learning, enhances exercise capacity and also mediates the acute stress response, the latter occurring independently of glucocorticoids (GC) but rather relying on the modulation of the parasympathetic nervous system.^71–77^ The endocrine effects of unOCN are believed to arise from its binding to GPR158 (in the CNS) and GPRC6A (in the periphery), both of which reflect 7-transmembrane helix receptors belonging to the G-protein coupled receptor superfamily.^64,78–81^

As for most (if not all) G-protein coupled receptors, the GPRC6A gene does not give rise to a single, but multiple receptor variants. To date, three such isoforms have been described. Posttranslational modification (i.e., 5′ alternative splicing) of the full-length mRNA generates two N-terminally truncated receptor variants, which are expressed rather poorly compared to the canonical isoform 1. Indeed, the full-length GPRC6A variant 1 is widely expressed among tissues with the highest abundance described in testis, brain and skeletal muscle.^81^ Subsequent scientific efforts culminated in the identification of cognate GPRC6A ligands thus yielding a deorphanization of the receptor.^82^ To date, several molecules have been suggested to bind and activate GPRC6A including basic amino acids (e.g., L-ornithine, L-arginine, L-lysine), divalent cations, testosterone and most notably, unOCN.^83^ While strong in vitro and in vivo evidence for an OCN-GPRC6A-axis exists,^71^ some authors have failed to reproduce direct binding of both OCN and testosterone to GPRC6A in cell-based assays.^84^ Given the wide distribution of GPRC6A expression and its multiple ligands that are highly abundant in most mammalian tissues, it remains to be deciphered if these molecules all compete for binding to the same receptor pocket, harness different binding sites and/or (hitherto unknown) coreceptors or perhaps exhibit varying (tissue-specific) affinities and potencies to engage and activate GPRC6A.

Collectively, the growing body of literature pointing toward essential functions of OCN in fueling diverse aspects of the fight and flight response gave rise to the concept of OCN reflecting a “survival hormone”.^74^ Yet, these observations also raise the question how such broad actions of a single mediator are fine-tuned and organized. Here, the theoretical framework of life history theory may help to shed new light on OCN biology.

### Endocrinology of unOCN and life history trade-offs

**Fig. 3:**
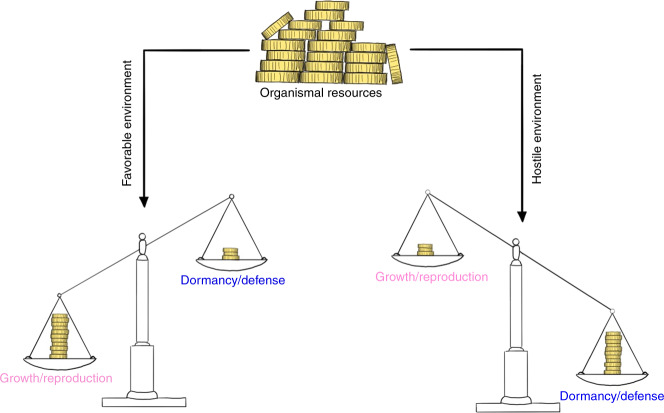
Life history trade-offs. Finite organismal resources have to be allocated to competing (opposing) biological programs through trade-offs. The underlying investment strategy is largely driven by environmental factors (life history theory). Whereas favorable environments promote resource allocation into growth and reproduction, hostile environments foster investments into dormancy and defense (collectively referred to as maintenance programs). This strict separation is crucial to maximize reproductive success and a common theme among life on earth

Each organism has a limited number of resources that can be allocated to different biological programs in order to maximize reproductive success. These investment strategies differ depending on the constraints imposed by the current environmental conditions encountered and can be roughly divided into four fields or two branches, respectively: reproduction and growth (branch 1), and dormancy and defense (branch 2, collectively referred to as “maintenance”). Whereas favorable environments (sufficient nutrient supply, few predators) promote the allocation of resources into branch 1, hostile environments elicit the opposite response (Fig. 3).^85,86^ The hostile nature of an environment may arise from either the absence of sufficient resources (typically, nutrients) or the presence of threats (such as predators, pathogens or toxins). Although dormancy and defense are both summarized by the umbrella term “maintenance”, they exhibit an inherently different nature tailored to the two types of hostile environments: defense programs are energetic costly (e.g., the inflammatory response to fight invading pathogens or the escape from predators), whereas dormancy programs (quiescence) are energy-preserving to permit survival in face of resource scarcity or other challenges (extreme temperatures etc.). Yet, both occur at the expense of growth and reproduction, which themselves impose a substantial energetic burden to the organism.^85^ Thus, an organized cellular as well as organismal orchestration of these biological programs is a common theme in biology (life-history trade-offs).

This concept may be exemplified by pathological conditions in humans such as hypothalamic amenorrhea or male exercising syndrome, where insufficient nutrient supply, over-exercising or psychological stress (mirroring ancient predators) result in a cessation of the hypothalamic-pituitary-gonadal axis (HPG), thus yielding suppressed sex hormone levels and in the presence of impaired fertility.^87,88^ From an evolutionary point of view, this adaptation (trade-off) is crucial because (a) insufficient nutrient supply or energy stores of the mother may leave the growing fetus with unmet demands and (b) the high energetic costs of sex hormone-driven biological responses should be avoided in light of such deficits.^89^ Similarly, the male individual should be able to provide nutrients to his offspring, although HPG axis in men appears to be somewhat less sensitive to stressors.

The necessity to separate these branches may be further illustrated by data derived from infectious disease models. When organisms are challenged with invading (bacterial) pathogens, they typically develop anorexia and sickness behavior as part of an adaptive defense program. When this response is perturbed through exogenous caloric supplementation, mortality increases, suggesting that forced shifting of one branch to another is detrimental to the host.^90^

**Fig. 4:**
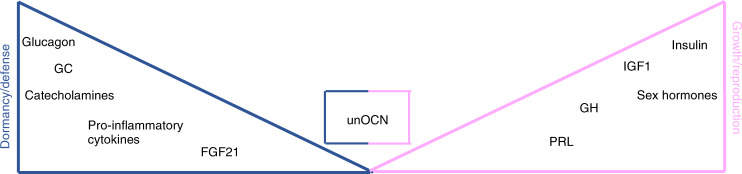
Endocrine hormones favor distinct biological programs. The balance between the two main life history branches (growth/reproduction vs. dormancy/defense) is heavily shaped by endocrine hormones and other mediators. While the latter do not exclusively promote either branch, their biology typically favors one over the other with varying nuances in between. This may be impressively exemplified by the antagonistic functions of glucocorticoids (catabolic, promote nutrient degradation, inhibit GnRH release) and insulin (anabolic, fosters nutrient storage, promotes GnRH release). Undercarboxylated osteocalcin (unOCN) actively contributes to both branches through its effects on fertility and the acute stress response. How these functions are fine-tuned and organized to avoid simultaneous activation of opposing biological responses remains to be determined. GC = glucocorticoids, GH = growth hormone, PRL = prolactin, IGF1 = insulin-like growth factor 1, FGF21 = fibroblast growth factor 21, unOCN = undercarboxylated osteocalcin

Although endocrine hormones and other mediators cannot be classifiable as exclusively mediating either growth/reproduction or dormancy/defense responses, their biology does typically favor one over the other (Fig. 4). There are many examples for this annotation: Glucocorticoids (GC) as prototypical stress hormones are mostly catabolic, effects predominantly arising from their ability to oppose anabolic functions. Consistently, cellular responses elicited by GC ultimately yield a breakdown of proteins, fatty acids as well as glycogen, thus providing fuel for organismal adaptations in face of various stressors.^91^ Of note, the same holds true for the other main class of stress hormones, namely catecholamines.^92^ At the organismal level, GC suppress HPG axis activity through inhibiting GnRH release,^93^ the latter reflecting a prioritization of energy distribution as described above. Of note, this response may become maladaptive if the stressor remains unresolved (e.g., infertility upon chronic stress exposure).

In contrast, insulin exerts broad anabolic effects, thereby promoting nutrient storage as well as tissue renewal and accrual.^94^ Consistently, insulin critically contributes to reproductive functions and growth.^95^

FGF21, a hepatokine induced upon fasting, exerts multiple effects such as increased ketogenesis, reduced growth as well as torpor, collectively reflecting energy-preserving mechanisms characteristic of dormancy programs.^96–98^ Moreover, FGF21 is a causal driver of starvation-induced hypogonadism, further underpinning its role as a mediator of such responses.^99^ Of note, additional functions of FGF21 in mammalian physiology have also been described. The latter have thus far only been partly understood and apparently exhibit a context-dependent fashion.^100^

IL1-beta holds distinct properties in defense programs as illustrated by its essential involvement in promoting inflammation, fever, cachexia and lethargy, all of which aid in fighting invading pathogens but occur at the expense of reproduction.^85,101^ Consistently, high levels of IL1-beta in obese individuals impair hypothalamic GnRH secretion, whereas IL1 antagonists (Anakinra) increase circulating testosterone levels in these patients.^102,103^

Finally, for testosterone, this separation may appear less sharp. While virtually all of the hormone’s effects can be considered anabolic (growth/reproduction), testosterone also supports some survival and defense functions (muscle growth/function, dominance/aggression, hematopoiesis). Yet, these contributions are of indirect, rather than direct nature. In fact, testosterone does not immediately participate in neither dormancy, nor defense. Although circulating levels of testosterone may rise upon acute stress exposure^104^ (which is, however, not unequivocally supported by scientific evidence^105^), castrated animals exhibit an exaggerated, rather than impaired CRH/ACTH response, suggesting that testosterone negatively regulates HPA axis activity.^106,107^ Similarly, observations from the animal kingdom have supported the concept that higher testosterone levels confer increased susceptibility to infectious disease through immunosuppressive effects arising from life-history trade-offs.^89^ Most importantly, unresolved environmental stressors uniformly provoke a cessation of the HPG axis and subsequent reductions in circulating testosterone levels, which is a conserved response across multiple species as outlined before.^108^

Osteocalcin (OCN), on the other hand, appears to actively contribute to both life-history branches by promoting both growth/reproduction (testosterone secretion by Leydig cells, insulin secretion and sensitivity, memory/learning) as well as dormancy/defense (mediation of the acute stress response).^71,72,74,76,109–111^ As such, OCN would contribute to maintaining fertility, high testosterone levels and muscle mass (i.e., anabolism), even in the face of hostile environments, reflecting a resource allocation conflict according to life history theory. These considerations not only hold true for stress and fertility but also for stress and glucose metabolism. Acute or prolonged stress is typically associated with insulin resistance to ensure sufficient glucose disposal to peripheral tissues.^112–114^ Along these lines, *Ocn*^*−/*^^−^ mice exhibited reduced circulating levels of glucose after exposure to stress, suggestive of impaired counterregulatory mechanisms.^74^ However, previously published data has suggested that *Ocn*^*−/*^^−^ mice are characterized by insulin resistance and obesity.^111^ As such, the lack of OCN apparently evokes opposing effects on glucose homeostasis under basal and stressful conditions, respectively. Taken together, the current experimental evidence points toward the intriguing concept that bone-derived OCN confers a significant evolutionary advantage to organisms through its pleiotropic effects that are essential to survival. Yet, how these functions are fine-tuned and organized to avoid simultaneous activation of opposing biological programs (life history trade-offs) remains to be deciphered.

It should be considered that the biology of most hormones differs between basal and “activated” states. As noted above, stress induced as well as iatrogenic glucocorticoid excess are associated with hypogonadism and sub-/infertility. On the other hand, physiological glucocorticoid receptor engagement supports certain reproductive functions such as spermatogenesis.^115^ Thus, OCN might belong to the same category of mediators with complex biology that participate in coordinating whole-body homeostasis in a context-dependent fashion.

### Challenges in translating OCN research to the clinic

Although a large body of evidence derived from genetic (as cited above), as well as pharmacological studies^116–118^ have underpinned many essential physiological functions of OCN in mouse physiology, the translational relevance of these findings for human diseases has remained unknown, with inter-species differences adding additional layers of complexity. In mice, two OCN encoding genes (Bglap-1 and Bglap-2,), as well as third osteocalcin-like gene (Bglpa-3) exist, the latter not being expressed in the skeleton. In contrast, humans and rats only exhibit a single OCN gene (BGLAP).^64,83^ Consistently, OCN is only moderately conserved across species with roughly 60% sequence homology between mouse and human Ocn, respectively,^83^ while the distal, functionally active regions appear to be mostly shared. However, the biological regulation of OCN expression also differs between the two species since vitamin D provokes an induction of OCN production in humans but elicits the opposite response mice.^119^ Similar unknowns exist for GPRC6A. Whereas murine Gprc6a is readily expressed on the cell surface membrane and functionally active, some studies have suggested that the human orthologue is largely retained in the cytoplasm. This biological discrepancy has been ascribed to two missing amino acid residues located in the third intracellular loop of the receptor.^120^ Contrary to these findings, knock-in of the human receptor orthologue in mice revealed positive effects on glucose homeostasis.^121^ Yet, the cellular location of the receptor’s expression was not reported by the authors, complicating the interpretation of these findings. In European and Asian populations, short (presumably, non-functional) GPRC6A variants appear to predominate, whereas African populations exhibit a higher frequency of longer variants.^120^ Given the relatively small sample sizes of the study, these findings need to be interpreted with caution. Nonetheless, some humans perhaps do not produce functional GPRC6A variants. On the other hand, several other studies have implicated that humans do express functionally active GPCR6A.^74,110,122^

Taking these observations together, one may ask why an endocrine system (OCN-GPRC6A-axis) regulating a variety of physiological functions of utmost importance including reproductive fitness (the latter reflecting the main driver of natural selection) is not evolutionary highly conserved across species as many other systems of equal (or perhaps even less) importance. For example, murine estrogen and µ-opioid receptors share 88% and 94% sequence homology with their respective human counterparts.^123,124^ Of note, the latter modulate ancient behaviors such as pain perception, produce a similar spectrum of splice variants across species and facilitate redundant cellular responses, underlining the evolutionary conserved advantage conferred by the implementation of this system.^124^ Consistently, other indispensable mediators such as steroid hormones are also conserved as exemplified by the identical structure of mouse and human testosterone. Future studies in vertebrates and (perhaps even more interesting) invertebrate species may deliver answers to these questions.

The transferability of OCN research to human diseases is further limited by the fact that no genome wide association study (GWAS) has thus far established a link between OCN and any of its suggested functions, although evidence of absence does likely not reflect absence of evidence in this scenario. Notably, GWAS have successfully been used to improve our understanding of diseases as well as developing novel pharmacological approaches. For example, in Crohn’s disease, such studies identified the IL23-axis as a potential pharmacological target with anti-IL23 antibodies now being used in the clinics.^125^ Mendelian Randomization (MR) studies, on the other hand, reflect a novel and even more robust approach to investigate the (causal) influence of a given trait (e.g., circulating OCN levels) on an outcome of interest (e.g., diabetes or hypogonadism). Indeed, such studies have uncovered a link between genetically determined circulating sclerostin levels and bone mineral density as well as fracture risk,^126^ although the original identification of sclerostin as a regulator of bone mass arose from genetic disorders, namely Van Buchem’s disease and Sclerosteosis.^127^ Consistently, anti-sclerostin antibodies such as romosozumab have proven effective in treating osteoporosis in patients,^128^ further underlining the validity of this tool. As such, the results of MR studies will help to better understand if the functions of OCN observed in mice are, in fact, extrapolatable to humans.

Notably, some genetic evidence does support a role of the putative OCN receptor GPRC6A in reproduction as illustrated by two studies linking mutations in GPRC6A to sub-/infertility in males.^110,122^ Although the significance of these studies is limited by their small sample sizes, experimental data suggests that these phenotypes may indeed be attributable to impaired OCN signaling.^122^ Yet, a potential involvement of other GPRC6A ligands cannot be definitely excluded at this point.

Recently, two studies reported no endocrine abnormalities in two novel *Ocn*^*−/−*^ mouse strains with respect to glucose homeostasis, testosterone levels and fertility. Of note, these mice were generated independently from each other using different methodologies (CRISPR/Cas9 and BAC modification technique, respectively).^129,130^ Both approaches targeted a similar chromosomal region as described in the original *Ocn*^*−/*^^−^ strain, thus yielding a loss of both murine Bglap genes. The reasons underlying the discrepancies between these studies and earlier reports are currently unclear. It is worth mentioning, however, that the phenotypic description of these animals revealed a rather inhomogeneous pattern characterized by large standard deviations. Moreover, the complexity of CRISPR/Cas9 gene editing holds a residual potential for off-target effects.^131^

As mentioned above, rat and human OCN share considerable higher homology compared to the murine locus. Consequently, researchers proposed that a generating an *Ocn*^*−/−*^ rat would produce a preclinical model more relevant to human physiology. Using a CRISPR-CAS9-based approach, the creation of such animals surprisingly did not reveal a striking metabolic phenotype. In fact*, Ocn*^*−/*^^−^ rats displayed an improved, rather than impaired insulin sensitivity. Conversely, similar skeletal alterations as in *Ocn*^*−/−*^ mice characterized by enhanced bone mass accrual were noted, suggesting that bone-intrinsic OCN biology might be conserved more strongly between the two species.^66^ Pharmacologically, treatment of SREBP1c transgenic rats with recombinant OCN had no apparent effect on glucose metabolism.^132^ On the other hand, recombinant OCN enhanced insulin secretion and glycemic control in a rat model of streptozotocin-induced type I diabetes, while the same parameters remained unaltered under homeostatic conditions.^133^

Lastly, conflicting data also exist on GPRC6A mouse models. In contrast to the widely used GPRC6A exon 2 knock-out mouse mimicking many phenotypic traits of *Ocn*^*−/−*^ animals,^71^ no such alterations were detected in other animals with complete disruption of the GPRC6A locus.^134^ While many of these unknowns remain to be investigated further, the observation that recombinant (un)OCN elicits a variety of biological responses and beneficially modulates diseases trajectories in preclinical models raises hope for a potential future role of OCN-based therapies in the clinic.

## Concluding remarks and future perspectives

The discovery of endocrine mediators produced by the skeleton has not only revolutionized our understanding of bone biology but also of endocrinology per se. For decades, textbooks and scientific publications have posited the classical concept of endocrine axis composed of the hypothalamus, the pituitary and an endocrine gland. Apparently, the skeleton integrates all three of these functions in a single organ, i.e., sensing (phosphate homeostasis), tropism (stimulation of testosterone synthesis by OCN) and glandular effector function (downregulation of NaP_i_s by FGF23).

With the development of Burosumab as the first FDA-approved anti-FGF23 antibody, the clinical translation of skeletal endocrine research has been gaining momentum. Although the use of Burosumab is currently restricted to X-linked hyperphosphatemia, future studies may resolve the crucial question whether pharmacological blockade of FGF23 elicits beneficial effects in highly prevalent diseases such as CKD or heart failure. For OCN, additional work is needed to comprehensively characterize its functions in humans in order to harness them for therapeutic purposes (see outstanding questions). Ultimately, targeting the GPRC6A-OCN-axis holds the promise of alleviating pathologies such as insulin resistance, immobility or hypogonadism, all of which impose a substantial health care burden in our aging society.

As such, the next chapter in skeletal endocrine research has only begun and new discoveries will certainly continue to enlighten this exciting scientific field.

## Outstanding questions

- Which biological functions can be attributed to C- and N-terminal FGF23, respectively?
- Does pharmacological blockade of FGF23 elicit beneficial effects in diseases such as heart failure or CKD?
- Which factors primarily drive FGF23 production in modern society?
- Why is cFGF23 production tightly linked to inflammation, iron deficiency and anemia? Does phosphate supplementation mimic the effects of enhanced cFGF23 production in these diseases?
- Are there additional layers of regulation in osteocalcin production/function that prevent simultaneous activation of opposing biological programs? If so, how is this accomplished?
- Does genetic and/or pharmacological elevation of circulating, unOCN levels yield enhanced insulin sensitivity, testosterone production and/or fertility?
- Do testosterone levels in men differentially respond to antiresorptive and osteoanabolic therapeutic regiments, respectively, and is this linked to changes in circulating unOCN?
- Are individuals with genetically determined higher unOCN serum levels superior athletes?
